# The rare coexistence of gastric and esophagus squamous cell carcinoma: a case report

**DOI:** 10.11604/pamj.2024.48.171.43878

**Published:** 2024-08-12

**Authors:** Yahya El Harras, Kaoutar Imrani, Sara Essetti, Ittimade Nassar, Nabil Moatassim Billah, Houda El Hiouy, Hicham El Bacha, Salma Mechhor, Manal Cherkaoui, Mariam Konso, Nadia Benzzoubeir, Ikram Errabih

**Affiliations:** 1 Department of Radiology, Ibn Sina University Hospital Center, Mohamed V University of Rabat, Rabat, Morocco,; 2 Department of Hepatogastroenterology and Proctology Medicine B, Ibn Sina University Hospital Center, Mohamed V University of Rabat, Rabat, Morocco

**Keywords:** Squamous cell carcinoma, esophagus, gastric, coexistence, case report

## Abstract

Squamous cell carcinoma (SCC) of the stomach is a rare entity with fewer than 100 cases of primary SCC reported in the literature, while esophageal SCC is prevalent and more common. However, a synchronous squamous cell carcinoma found in the esophagus and stomach remains very uncommon. We present the case of a 64-year-old with a history of dysphagia who had an endoscopy that showed an impassable stenosis of the middle esophagus, with histopathology in favor of an esophagus squamous cell carcinoma. A computed tomography scan (CT-SCAN) then showed an exophytic mass of the lesser curvature of the stomach with the biopsy in favor of a squamous cell carcinoma. Our case report aims to keep in clinicians´ and anaomopathologists´ minds that esophageal SCC may coexist with gastric SCC and that the role of imaging is important in the diagnostic procedure.

## Introduction

It is commonly recognized that most gastric malignancies (about 95%) are adenocarcinomas while primary gastric squamous cell carcinomas (SCC) are extremely rare [1,2]. Thus far, fewer than 100 primary gastric SCCs have been documented [3]. On the other hand, squamous cell carcinomas are prevalent in the esophagus. Although gastric adenocarcinomas and esophageal squamous cell carcinomas have been reported simultaneously in a few cases, a synchronous SCC found in the esophagus and stomach is uncommon and has not been reported in Morocco, yet. We are presenting a 64-year-old female who presented with dysphagia with Esophagogastroduodenoscopy (EGD) showing an impassable stenosis of the middle esophagus and histopathology in favor of an esophagus squamous cell carcinoma. Thoracic and abdominopelvic CT-scan then showed an exophytic mass of the lesser curvature of the stomach and its biopsy was in favor of a coexistent squamous cell carcinoma. Clinicians, anatomical pathologists, and radiologists should be aware that the coexistence of gastric and esophagus squamous cell carcinoma is possible.

## Patient and observation

**Patient information:** a 64-year-old woman with no history of smoking, alcohol consumption, or previous surgery, presented to the gastroenterology department with dysphagia to solid food.

**Clinical findings:** clinical examination on admission found a moderately dehydrated patient with a Body Mass Index (BMI) of 17 kg/m^2^. Pallor in conjunctivae and epigastric sensibility were also noted.

**Timeline of the current episode:** complaints of dysphagia started three months ago, accompanied by postprandial back radiating and epigastric pain. These symptoms were associated with loss of appetite and weight loss estimated at 13kg.

**Figure 1:**
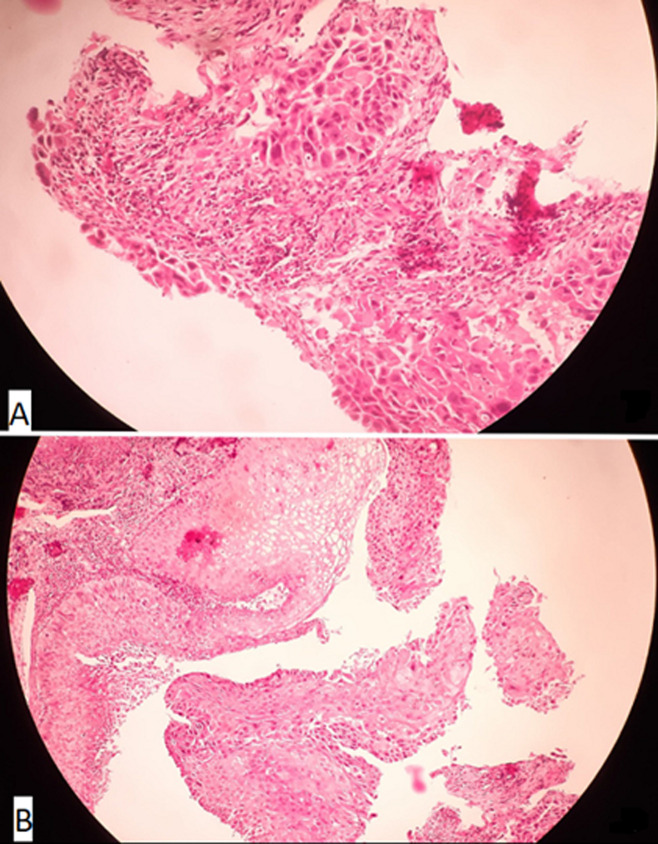
esophagus mass histopathology: squamous cell; A) hematoxylin-eosin staining x10; B) hematoxylin-eosin staining x20 made of atypical squamous cells with invasion through the basement membrane, prominent central nucleus, dense-appearing eosinophilic cytoplasm and squamous whorls

**Figure 2:**
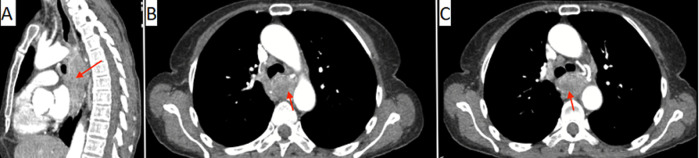
A,B,C) sagittal and axial images of the thoracic computed tomography scan with intravenous contrast showing the esophageal mass (red arrow) obstructing the lumen and presenting contact with the trachea and its two main bronchi

**Figure 3:**
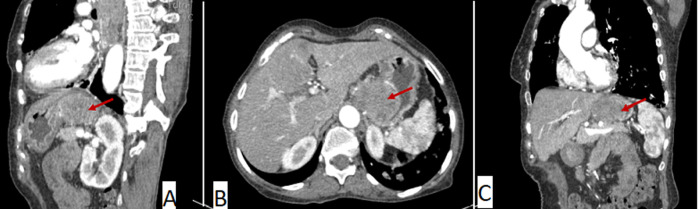
A) sagittal; B) axial; C) coronal images of the abdominal computed tomography scan showing the tissular stomach mass, heterogenous with exophytic development (red arrow)

**Diagnostic assessment:** esophagogastroduodenos copy (EGD) showed a red irregular round and plate-like lesion with raised borders at the mid-esophagus. The mass was obstructing the lumen; thus, the clinicians performed a biopsy but could not explore further. Histopathological examination showed typical elements of squamous cell carcinoma (Figure 1). The patient was then referred to our department for a thoracic and abdominopelvic CT scan to assess distant metastases. Our scan showed in addition to the mass extending in the posterior wall of the thoracic esophagus, a tissular wall mass of the lesser curvature of the stomach with an exophytic development (Figure 2, Figure 3). An association of a gastrointestinal stromal tumor (GIST) with an esophagus squamous cell carcinoma was suspected. We realized an ultrasound-guided biopsy of the stomach mass for the patient.

**Figure 4:**
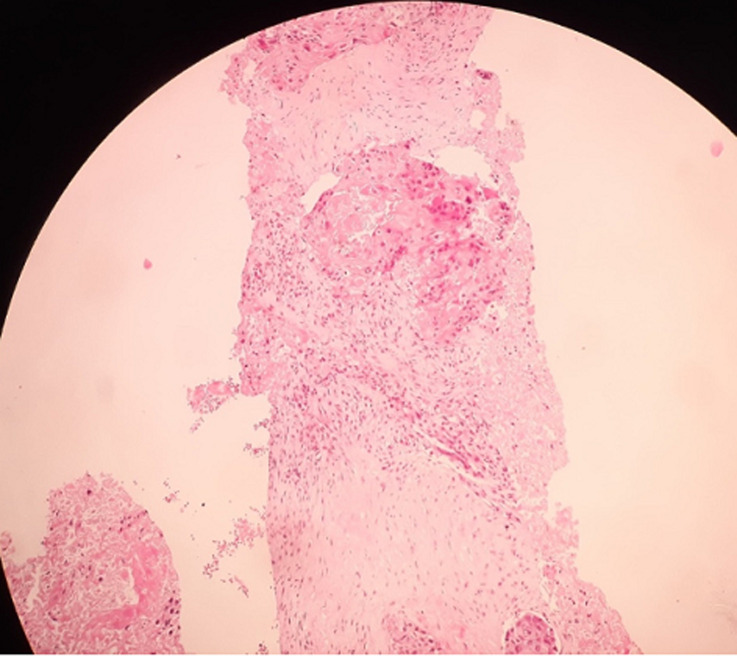
gastric mass histopathology: connective tissue infiltrated by carcinomatous proliferation made of nests and solid masses of arranged polygonal cells showing obvious cytonuclear atypia, in favor of a mature, well-differentiated keratinizing squamous cell carcinoma of the stomach

**Diagnosis:** histopathological examination showed elements in favor of a mature, well-differentiated keratinizing squamous cell carcinoma of the stomach (Figure 4). Thus, the diagnosis of a coexistent esophageal and gastric SCC was confirmed.

**Therapeutic interventions:** gastroenterologists performed an endoscopic dilation of the esophageal lumen and scheduled the patient for a multidisciplinary team meeting to discuss treatment options.

**Follow-up and outcome of interventions:** unfortunately, the patient passed away due to respiratory distress in the intensive care unit five days later after the procedure.

**Informed consent:** our institution does not require ethical approval for reporting individual cases or case series. However, written informed consent was obtained from a legally authorized representative for anonymized patient information to be published in this article.

## Discussion

A synchronous SCC developing in the esophagus and stomach is extremely uncommon among gastrointestinal cancers [4]. Squamous cell carcinoma is prevalent in the esophagus, accounting for 38% of all esophageal malignancies but rare in the stomach. It mainly occurs in the mid to lower esophagus, and it is not rare to coexist with other cancerous lesions with the stomach being the most common location, followed by the colon and rectum [4]. Furthermore, it has been noted that the mortality from the cancerous lesions that accompany esophageal cancer is higher than the mortality from the cancer itself [5]. On the other hand, primary gastric SCC accounts for only 0.04-0.5% of cases of all gastric cancer cases [6]. Diagnostic criteria for primary gastric SCC were first described in 1967, requiring three features [7]: a) the tumor cannot be at the cardia; b) it cannot spread into the esophagus; and c) there must be no indications of squamous cell carcinoma (SCC) in any other body area. In 2011, the Japanese Gastric Cancer Association revised it and put out criteria that included the following: i) It is necessary for all tumor cells to be SCC cells, devoid of any gland cancer cells; and ii) Squamous cell carcinoma must originate in the stomach mucosa [8].

Though the exact pathogenesis of stomach squamous cell carcinoma (SCC) is unknown, some theories have been proposed to explain its development, such as squamous differentiation in a preexisting adenocarcinoma or squamous metaplasia of the gastric mucosa before malignant transformation [9]. Also, ectopic squamous epithelium appears to be extremely rare in the stomach. Still, squamous metaplasia has been described in healing gastric ulcers and a variety of conditions associated with long-standing chronic inflammation [10]. Furthermore, some cases of gastric SCC may have an Epstein-Barr virus infection as a contributing factor in their etiology [10]. In our case, there was no evidence of either squamous metaplasia or ectopic squamous mucosa. In addition, we were unable to locate any glandular components within the tumor and there was no evidence of Helicobacter or viral infection. Endoscopy is regarded as the most sensitive and specific diagnostic method in patients suspected of having gastric or esophageal cancer. It allows direct visualization of the tumor´s location, the extent of mucosal involvement, and biopsy for histopathological diagnosis. However, imaging methods are often the initial tool that raises suspicion for gastric carcinoma, besides being used in the staging of the disease. On a CT scan, a tissue wall thickening obstructing the esophagus lumen may be seen with or without mediastinal invasion.

A mass effect on the posterior wall, stenosis, and tracheobronchial displacement may also be present. As for gastric cancers, the main signs are a polypoid mass with ulceration, focal wall thickening, irregularity of the mucosa, or focal infiltration of the wall. Gastric SCC can rarely present as a submucosal mass and should be differentiated from other neoplasms, including gastrointestinal stromal tumors (GIST), carcinoids, melanomas, and lymphomas with the most common submucosal tumors being GIST as we know [3,10]. On CT-scan, SCC of the stomach usually shows a heterogeneously enhanced mass. Huge GIST can show the same aspect, due to necrosis, hemorrhage, or degenerative components [10]. Gastroscopic mucosal biopsy could help in identifying those submucosal masses. However, a preoperative biopsy is not recommended for tumors highly suspected of GIST and could be completely removed [10]. Still, many studies have supported the idea of preoperative biopsy even in the case of GIST suspicion, due to the importance of the preoperative diagnosis in deciding the surgical method [3]. Such was the case with our patient, since he had an esophageal mass, obstructing the lumen. Endoscopic ultrasound-fine needle biopsy is also an alternative that can be considered when no abnormalities are found in the first biopsy.

For locally advanced esophageal cancer, the American Society of Clinical Oncology (ASCO) recommends a treatment plan that combines different types of treatments. In the case of SCC, chemoradiotherapy is commonly recommended as the first treatment and surgery may be used afterward [9]. A standard treatment plan for gastric SCC has not yet been established, although its clinical symptoms and epidemiological characteristics have been reported in the literature. It is debated whether or not to use the existing therapeutic approaches for gastric adenocarcinoma (ADC) in the management of gastric SCC, due to differences in molecular characteristics, tissues of origin, and prognosis. When it comes to gastric adenocarcinoma, there is no doubt that surgery remains the only potentially curative treatment [10]. In the case of gastric cancer involving the fundus and/or the body of the stomach, most surgeons perform total gastrectomy [7]. The dilemma remains whether to manage gastric SCC according to the therapeutic principles of esophageal SCC or that of gastric ADC. Consequently, treating coexistent esophageal and gastric squamous cell carcinomas has not been reported to this date.

## Conclusion

Our case report illustrates that synchronous cancers can be discovered with esophageal squamous cell carcinoma. Therefore, physicians, anatomical pathologists, and radiologists should be aware that there may be potential cancer lesions other than the esophagus while establishing a diagnosis, staging a cancer, and assessing therapy response.

## References

[1] Gülçiçek OB, Solmaz A, Özdoğan K, Erçetin C, Yavuz E, Yiğitbaş H (2015). Primary squamous cell carcinoma of the stomach. UCD.

[2] Dong C, Jiang M, Tan Y, Kong Y, Yang Z, Zhong C (2016). The clinicopathological features and prognostic factors of gastric squamous cell carcinoma. Medicine (Baltimore).

[3] Gao L, Tang X, Qu H, He Q, Sun G, Shi J (2020). Primary gastric squamous cell carcinoma presenting as a large submucosal mass: a case report and literature review. Medicine (Baltimore).

[4] Lim PS, Greenberg M, Edelson MI, Bell KA, Edmonds PR, Mackey AM (2011). Utility of ultrasound and MRI in prenatal diagnosis of placenta accreta: a pilot study. AJR Am J Roentgenol.

[5] Sato Y, Motoyama S, Maruyama K, Okuyama M, Ogawa J (2005). A second malignancy is the major cause of death among thoracic squamous cell esophageal cancer patients negative for lymph node involvement. J Am Coll Surg.

[6] Akce M, Jiang R, Alese OB, Shaib WL, Wu C, Behera M (2019). Gastric squamous cell carcinoma and gastric adenosquamous carcinoma, clinical features and outcomes of rare clinical entities: a a National Cancer Database (NCDB) analysis. J Gastrointest Oncol.

[7] De Lange G, Bouroumeau A, Coron E, Koessler T (2023). Gastric squamous cell carcinoma: a rare malignancy, literature review and management recommendations (Review). Mol Clin Oncol.

[8] Guzman Rojas P, Parikh J, Vishnubhotla P, Oharriz JJ (2018). Primary Gastric Squamous Cell Carcinoma. Cureus.

[9] Japanese Gastric Cancer Association (2011). Japanese classification of gastric carcinoma: 3^rd^ English edition. Gastric Cancer.

[10] Callacondo-Riva D, Ganoza-Salas A, Anicama-Lima W, Quispe-Mauricio A, Longacre TA (2009). Primary squamous cell carcinoma of the stomach with paraneoplastic leukocytosis: a case report and review of literature. Hum Pathol.

